# Biomechanical and clinical research of Isobar semi-rigid stabilization devices for lumbar degenerative diseases: a systematic review

**DOI:** 10.1186/s12938-023-01156-1

**Published:** 2023-09-23

**Authors:** Jianbin Guan, Tao Liu, Xing Yu, Wenhao Li, Ningning Feng, Guozheng Jiang, He Zhao, Yongdong Yang

**Affiliations:** 1 Department of Spine Surgery, Honghui-Hospital, Xi’an Jiaotong University, Xi’an, 710054 China; 2 Dongzhimen Hospital, Beijing University of Chinese Medicine, Beijing, 100700 China

**Keywords:** Isobar TTL, Adjacent segment disease, Range of motion, Posterior stabilization, Systematic review

## Abstract

While lumbar spinal fusion using rigid rods is a prevalent surgical technique, it can lead to complications such as adjacent segment disease (ASDis). Dynamic stabilization devices serve to maintain physiological spinal motion and alleviate painful stress, yet they are accompanied by a substantial incidence of construct failure and subsequent reoperation. Compared to traditional rigid devices, Isobar TTL semi-rigid stabilization devices demonstrate equivalent stiffness and effective stabilization capabilities. Furthermore, when contrasted with dynamic stabilization techniques, semi-rigid stabilization offers improved load distribution, a broader range of motion within the fixed segment, and reduced mechanical failure rates. This paper will review and evaluate the clinical and biomechanical performance of Isobar TTL semi-rigid stabilization devices. A literature search using the PubMed, EMBASE, CNKI, Wanfang, VIP, and Cochrane Library databases identified studies that met the eligibility criteria. Twenty-eight clinical studies and nine biomechanical studies were included in this systematic review. The VAS, the ODI, and Japanese Orthopedic Association scoring improved significantly in most studies. UCLA grading scale, Pfirrmann grading, and modified Pfirrmann grading of the upper adjacent segments improved significantly in most studies. The occurrence rate of ASD was low. In biomechanical studies, Isobar TTL demonstrated a superior load sharing distribution, a larger fixed segment range of motion, and reduced stress at the rod–screw/screw–bone interfaces compared with titanium rods. While findings from mechanical studies provided promising results, the clinical studies exhibited low methodological quality. As a result, the available evidence does not possess sufficient strength to substantiate superior outcomes with Isobar semi-rigid system in comparison to titanium rods. To establish more conclusive conclusions, further investigations incorporating improved protocols, larger sample sizes, and extended follow-up durations are warranted.

## Introduction

Spinal surgery includes three main components to reduce pain and disability: decompression, stabilization, and deformity correction. Various pathologic conditions require combinations of these procedures. Lumbar fusion embodies these processes in a concentrated manner, and it is recognized as the “gold standard” for treating lumbar degenerative diseases. The primary objective of fusion is to achieve a solid connection between the specified segments, establishing rigidity. The mechanical properties of the implant material have a significant impact on the quality and efficacy of a fusion. Titanium rods provide the spine a lot of rigidity, which increases the fusion rates. However, previous studies have thoroughly discussed the disadvantages of titanium rods [1, 2], such as over-stabilization [3] and stress shielding [4], which leads to adjacent segment degeneration. As a result, non-rigid stabilization and motion preservation techniques have advanced quickly [5].

Pedicle screw-based dynamic stabilization (PDS) devices, as opposed to the rigid rods commonly employed in standard instrumented fusion, employ motion-preserving constructs that intricately link pedicle screw fixations [6]. Originally conceived to stabilize the aberrant segment and alleviate strain on degenerated discs and facet joints, these devices strive to uphold the natural curvature of the spine. By unloading the pressure on the degenerated disc and facets, these devices have the potential to reduce pain associated with anatomical structures. Furthermore, these devices have the potential to prevent adjacent segment disease (ASDis). This can be accomplished by substituting the entire construct with dynamic rods or implementing a "topping-off" strategy on the rigid instrumented segment. This strategy avoiding sudden increase in load from a rigid construct to the adjacent anatomical structures [7–9]^.^ It has also been argued that using PDS devices can improve fusion by allowing for micromovements between endplates and prevent against implant failure through improved load sharing [10, 11]. So far, various PDS systems have been described in the literature, encompassing semi-rigid rod systems used primarily for fusion and tension band-based posterior systems generally used as no-fusion technologies [12–16].

**Fig. 1:**
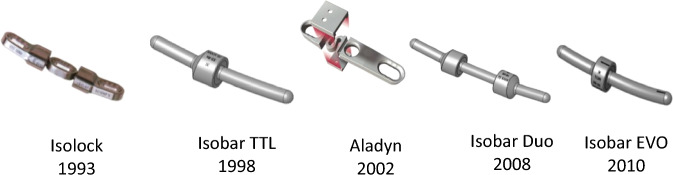
Evolution of 5 generations of Isobar systems

**Fig. 2:**
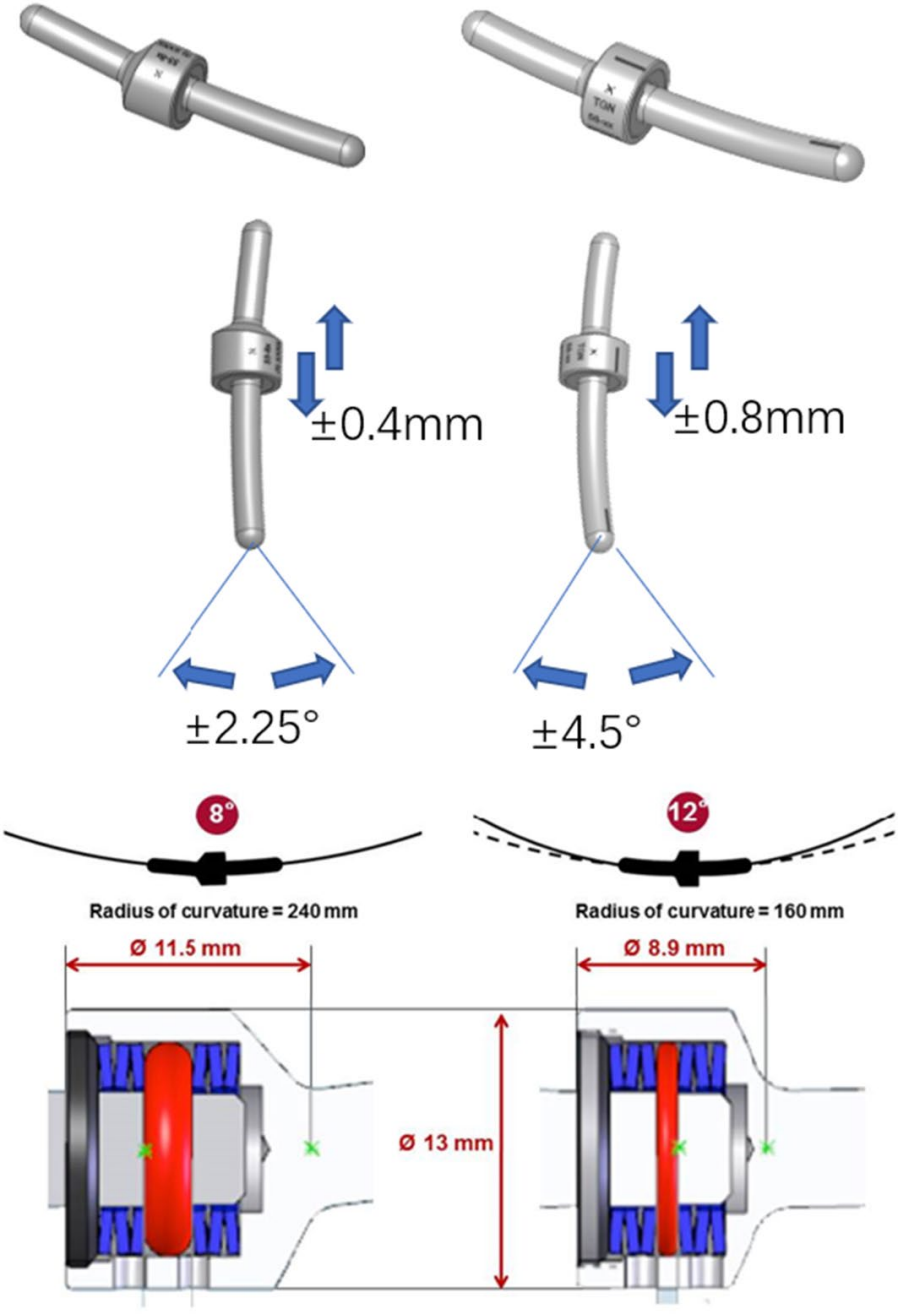
Isobar dynamic stabilization devices (Isobar TTL, Left, Isobar EVO, Right)

**Fig. 3:**
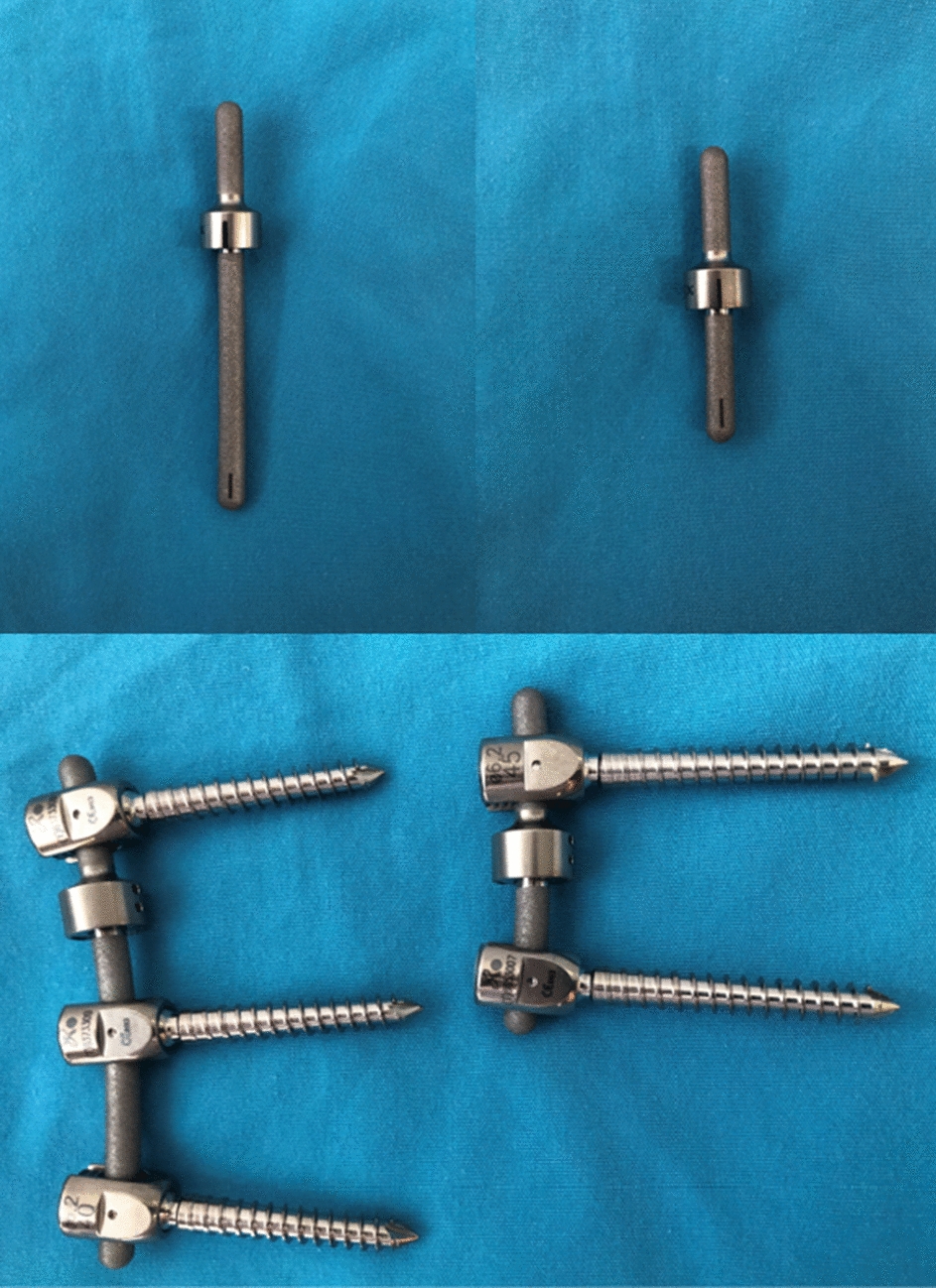
Isobar dynamic stabilization devices (Isobar EVO; Hybrid Left, single level Right)

Semi-rigid devices generally consist of metallic rods using hinges, springs, or bumpers to allow for partially controlled 3-dimensional motion or micromotion, such as Isolock, Isobar TTL, Isobar EVO, and PEEK rod. Isobar semi-rigid rod and PEEK rod systems stand out as notable examples. However, the systematic evaluation of Isobar semi-rigid system has been absent from the literature. In this article, we aim to address this gap by providing a detailed description of Isobar semi-rigid system through this systematic review. Isobar semi-rigid system theoretically have better biomechanical and clinical advantages. This system has undergone evolutionary iterations including Isolock, Aladyn, Isobar Duo, Isobar TTL, and Isobar EVO, with the TTL system being the most widely used (Fig. 1). As dynamic stabilization, flexible stabilization, or semi-rigid fixation methods for spinal stabilization and fusion have become more popular, Isobar system has become a promising candidate material [17]. This system can achieve a critical balance between sufficient stabilization and symptom relief, mediated by a reasonable loading distribution and the reduced interruption of physiologic motion that reduces the risk of ASDis. The Isobar system consists of a metallic semi-rigid PDS device made of titanium (minimum artifacts on MRI and CT). It contains a damper component in its longitudinal element, an 11.5 mm (TTL) or 8.9 mm (EVO) titanium alloy rod. The damper, i.e., the dynamic component, allows reduced stiffness and limited amount of angular and axial micromotion. The damper provides ± 2.25° (TTL) or ± 4.5° (EVO) angular ROM in flexion–extension and lateral bending, no limitation in axial rotation (unconstrained) and ± 0.4 mm axial ROM (TTL) or 0.8 mm axial ROM (EVO). (Figs. 2 and 3).

Here, we performed a systematic review to collect and analyze all data available regarding the clinical and biomechanical evaluation of Isobar semi-rigid system in lumbar spinal fusion, non-fusion and Hybrid surgery.

## Results

### Study selection

The process we employed for identifying and analyzing studies is shown in Fig. 8. A total of 1195 articles were identified from 6 databases (459 articles from PubMed, 486 from EMBASE, 89 articles from CNKI, 76 articles from Wanfang, 91 articles from CAJ, and no articles from Cochrane). After removing the duplicates, 603 articles were left for abstract review. We excluded 551 additional articles for obvious irrelevance, leaving 52 studies for title and abstract review, and underwent a comprehensive full-text review. Finally, 40 studies met the eligibility criteria. Data were extracted and the quality of each individual study was assessed.

### Quality assessment

**Fig. 4:**
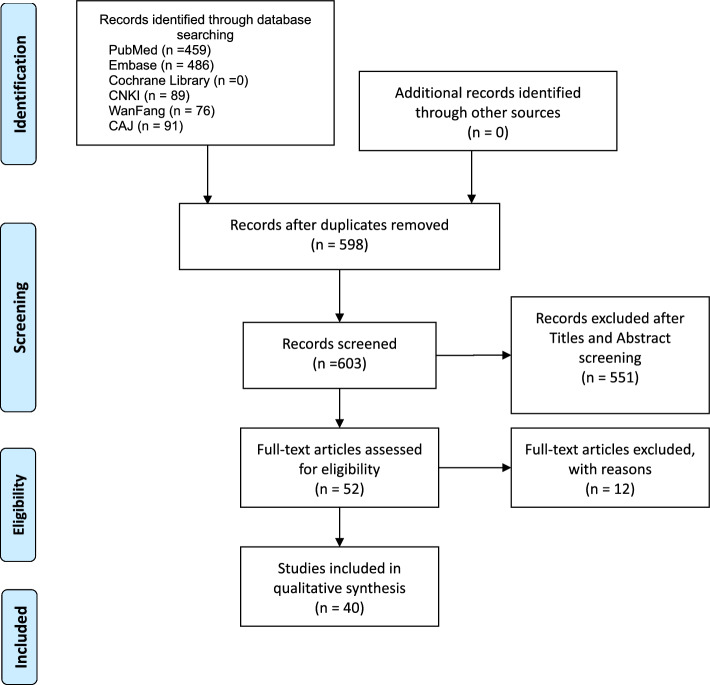
Flow diagram of the study selection process

In this systematic review, we wanted to collect all the available information about the clinical applications and biomechanical properties of Isobar semi-rigid system. The majority of the available clinical studies were retrospective cohort design and therefore had low quality of evidence grades according to our grading system. Four RCTs were assessed to have low risk of bias due to methodological quality based on Cochrane risk-of-bias criteria (the quality of the four included RCTs is shown in Table 1 and Fig. 4). And 19 studies were assessed to have medium risk of bias due to methodological quality based on their NOS score of six or seven possible points. The remaining eight papers were assigned NOS scores of eight possible points, indicating low risk of bias. The quality of the four included observational studies is shown in Table 2. The GRADE system considers strength of recommendation is strong. No biomechanical study could be assigned an evidence level.

**Table 1:** Study quality of included RCT on the Cochrane risk-of-bias criteria

RCT	Random sequence generation	Allocation concealment	Blinding of participants and personnel	Blinding of outcome assessment	Incomplete outcome data	Selective reporting	Other bias
Deng et al. [21]	Low risk	Low risk	High risk	Unclear risk	Unclear risk	Low risk	Unclear risk
Feng et al. [28]	Low risk	Low risk	Unclear risk	Low risk	Unclear risk	Low risk	Unclear risk
Liu et al. [41]	Low risk	Low risk	Unclear risk	Low risk	Unclear risk	Low risk	Unclear risk
Gao et al. [45]	Low risk	Low risk	High risk	Unclear risk	Unclear risk	Low risk	Unclear risk

Other bias: the baseline characteristics in the experimental and control groups were different

Low quality: either the randomization sequence generation or the allocation concealment was graded as high or unclear risk, regardless of the risk of the other items

High quality: both the randomization sequence generation and the allocation concealment were graded as low risk, and all the other items except the blinding of participants and personnel were assessed of low or unclear risk

Moderate quality: not meeting the criterion of high and low quality

**Table 2:** Study quality of included cohort studies based on the Newcastle–Ottawa scale

Authors	Representativeness of the exposed cohort	Selection of the non-exposed cohort	Ascertainment of exposure	Demonstration that outcome of interest was not present at start of study	Comparability of cohorts on the basis of the design or analysis^a^	Assessment of outcome	Was follow-up long enough for outcomes to occur	Adequacy of follow-up of cohorts	Scores
Qian et al. [20]	★	★	★	★	★	★	★	★	8
Huang et al. [22]	★	★	★	★	★★		★		7
Tian et al. [23]	★		★	★	★		★	★	6
Yang et al. [24]	★	★	★	★		★	★	★	7
Wen et al. [25]	★	★	★	★	★		★	★	7
Zhang et al. [26]	★		★	★	★	★	★	★	7
Lu et al. [27]	★	★	★	★		★	★	★	7
Zeng et al. [29]	★	★	★	★	★	★	★	★	8
Cao et al. [30]	★		★	★	★★		★	★	7
Huang et al. [31]	★	★	★	★	★	★	★	★	8
Xu et al. [32]	★		★	★	★		★	★	6
Ma et al. [33]	★		★	★	★★		★	★	7
Liu et al. [34]	★	★	★	★	★	★	★	★	8
Rao et al. [35]	★	★	★	★		★	★	★	7
Yao et al. [36]	★	★	★	★	★	★		★	7
Li et al. [37]	★	★		★	★	★	★	★	7
Cedric Barrey et al. [38]	★	★	★	★	★	★	★	★	8
Zhang et al. [39]	★	★	★	★	★		★	★	7
Liu et al. [40]	★	★	★	★		★	★	★	7
Ji et al. [42]	★		★	★	★		★	★	6
Song et al. [43]	★		★	★		★	★	★	6
Zhang et al. [44]	★	★		★	★★		★	★	7
Li et al. [46]	★		★	★		★	★	★	6
Rong et al. [47]	★	★		★	★	★		★	6
Guan et al. [48]	★	★	★	★	★	★	★	★	8
Guan et al. [49]	★	★	★	★	★	★	★	★	8
Zhao et al. [50]	★	★	★	★	★	★	★	★	8

^a^A maximum of 2 stars can be allotted in this category, one for age, the other for other controlled factors

## Clinical studies

Thirty-one clinical studies were included in this systematic review, 27 of which were retrospective studies and 4 were randomized controlled trials.

### Fusion surgery and Hybrid surgery

Three studies [27, 29, 38] reported the fusion surgery with Isobar semi-rigid system. Lu et al. [27] and Zeng et al. [29] presented case series discussing the use of Isobar TTL in single-segment isthmus bone grafting fusion technique. Lu et al. [27] assessed 49 patients diagnosed with lumbar spondylolysis or with degree I spondylolisthesis that resulted in some form of instability associated with neurogenic or radicular pain or chronic back pain. These patients underwent a laminectomy or discectomy with pedicle screw fixation using Isobar TTL with isthmus bone grafting. In a similar vein, Zeng’s series study encompassed 26 cases. Both studies used patient self-reporting parameters, including the VAS and ODI, and reported the fusion rate. Lu et al. [27] found that the VAS and ODI were both significantly improved after surgery and with the 85.71% and 97.96% fusion rate at 6 months and 12 months after surgery. Zeng et al. [29] also found a significant improvement in the VAS an ODI, and the fusion rate in his study is 88.5% (23/26) at final follow-up. Furthermore, they also explored the upper adjacent segment of the surgical segment and discovered that there was no significant difference in the UCLA classification of the disc before surgery and after surgery. As a result, the authors convinced that Isobar TTL would be more effective in preventing adjacent segment degeneration. Cedric Barrey et al. [38] offers a long-term (average 10.2 years) insight view of the clinical outcomes if 18 patients were treated by single or double segments interbody fusion with Isobar TTL for degenerative lumbar spine diseases. All patients returned to work except for two cases that retired during follow-up. 15/18 were entirely satisfied with the treatment. And for solid fusion, was observed 16/18 (89%) for dynamic procedure and uncertain in 2 cases with outcomes for these patients good and excellent, respectively. The findings outline significant and stable symptoms relief, absence of implant-related complications, no revision surgery, and few adjacent segment degenerative changes.

Sixteen articles [24–26, 28, 30–32, 34–36, 39, 42, 44–46, 49] compared Isobar TTL applied to multi-segmental Hybrid surgery with titanium rod multi-segmental fusion for comparison. Feng et al. [28], Cao et al. [30], Gao et al. [45], and Guan et al. [49] were compared between groups and before and after surgery for upper adjacent segmental disc degeneration using UCLA classification, modified Pfirrmann grading, and Pfirrmann grading, respectively. The authors discovered that each grade in the TTL group was significantly lower than that in the Ti group.

### *Non-fusion surgery and ROM *in vivo

Most authors reported using Isobar semi-rigid system for lumbar non-fusion surgery. Four retrospective cohort studies [20, 22, 23, 47] reported patients underwent single-level posterior lumbar decompression and Isobar TTL dynamic internal fixation for degenerative lumbar spinal disease with or without Meyerding I spondylolisthesis for a chief complaint of axial back pain. The authors concluded that this surgery technique can effectively relieve pain, improving quality of life, such as VAS, ODI, and JOA scores. And radiographic measurements such as the range of motion (ROM), UCLA classification of upper adjacent segment, and the disc height (DH) were calculated. Qian et al. [20] discussed the ROM of the fixed segment. The authors observed that the preoperative ROM was 3.46°, while the ROM at 12 months after the operation was 2.25°. However, the disparity between these values was not found to be statistically significant. In contrast, through the evaluation of fixed segment mobility before and after surgery in a group of 20 patients, Tian et al. [23] reported a noteworthy reduction in mobility. Specifically, the mobility diminished from 4.5° before the surgery to 2.5° after the surgery (*p* < 0.05). Huang et al. [22] and Tian et al. [23] also found that there was no significant difference between the preoperative and postoperative conditions. This conclusion was drawn by comparing the mobility of adjacent segments and the Disc Height (DH) of both the fixed segment and the adjacent segment. Furthermore, both Tian et al. [23] and Rong et al. [47] reported that the UCLA classification of the upper adjacent segment showed no significant difference between pre-operation and the final follow-up assessment. Similarly, Huang et al. [22] observed that the nucleus pulposus volume (NVP) of the upper adjacent segment exhibited no significant difference between the preoperative stage and the 48-month postoperative evaluation. In the context of unilateral single-level non-fusion cases utilizing the Isobar TTL system, Ma et al. [33] noted an increase in ROM of the adjacent segment from 2.93° to 5.18°, while the ROM of the fixed segment decreased from 2.85° to 2.33° (*p* < 0.05). Moreover, both the UCLA grading scale and the modified Pfirrmann grading displayed no statistical significance between the preoperative and postoperative periods.

In several studies [18, 33, 36, 37], the authors compared the Isobar TTL system with Dynesys, titanium rods, lumbar discectomy (LD), and percutaneous endoscopic lumbar discectomy (PELD). Deng et al. [21] investigated the outcomes of 60 patients treated with the Isobar TTL system in comparison to another 60 patients treated with the Dynesys system. The author noted a notable enhancement in both VAS and ODI scores. However, it was found that the Dynesys system exhibited superior effectiveness in maintaining segmental motor function (4.8° vs. 2.8°). Li et al. [37] reported the Isobar TTL outperforms titanium rod in terms of ROM of lumbar and fixed segment retention. Xu et al. [32] conducted a comparative analysis of outcomes between 20 patients who underwent the TTL procedure and another 20 patients who received traditional titanium rods. The results revealed no statistically significant disparities in clinical outcomes between the 2 groups. When lumbar mobility was compared, the TTL group was significantly higher than the titanium rod group, and there was no difference in lumbar mobility before and after surgery in the TTL group, whereas there was a significant difference in the titanium rod group (TTL group: 14.72° at pre-op, 13.92° at final follow-up; Ti group: 13.55° at pre-op, 7.34° at final follow-up). When compared with LD or PELD, both Liu et al. [34] and Liu et al. [40] reported that the TTL group achieved better clinical and radiological outcomes. Moreover, in the TTL group, the ROM in the lumbar region as well as the fixed segment was notably higher compared to the PELD group after the surgery. Additionally, the TTL group exhibited a lower increase in compensatory mobility in the upper adjacent segment in comparison to the LD group. For non-fusion revision surgery, Song et al. [43] performed non-fusion fixation of the problem segment using the Isobar EVO system in 15 patients presenting with upper ASD due to titanium rod fusion. The authors reported a significant improvement in the VAS and ODI. Although the average range of motion (ROM) of the fixed segment decreased from 6.32° prior to surgery to 3.16° at the final follow-up, the mean ROM of the adjacent segment increased from 4.87° before the surgery to 5.51° at the final follow-up. Furthermore, the lumbar lordosis (LL) increased from 27.12° preoperatively to 30.95° at the final follow-up. Despite these changes, the disc height index at the adjacent segment did not exhibit a statistically significant difference compared to the preoperative values. Over the course of the follow-up period, no cases experienced internal body loosening, and there were no instances of recurrent adjacent segment disease.

The pelvic parameter may affect outcomes such as pain relief and posture improvement after surgery, thereby influencing patient satisfaction and quality of life. Two authors focused on lumbar lordotic angle (LL) and sacral slope angle (SS) specifically. Yang et al. [24] discussed the results of 52 patients who received Isobar TTL compared with another 46 patients who received the Titanium rods. The authors reported a significant improvement in the VAS and JOA results in both fusion groups. And the LL and SS significantly different between the two groups, and the TTL group with a larger angle than the Ti group (22.8° vs. 13.9°, 15.9° vs. 12.2°). Huang et al. [22] reported the results of a 74-patient cohort study in which 36 patients underwent Isobar TTL Hybrid and 38 patients received posterior autologous grafting with titanium rods. There were no statistically significant clinical outcome differences between the two groups, which was scored with JOA, but at the 2-year follow-up the LL and SS significantly different between the two groups (20.5° vs. 14.1°, 15.8° vs. 12.3°).

### ASD prevention and complications

Six studies [24, 25, 31, 42, 48, 50] specifically addressed the incidence of ASDis. Yang et al. [24], Wen et al. [25], Huang et al. [31], and Ji et al. [42] reported 3.8% (2/52), 2.7% (1/36), 2.7% (1/36), and 5% (1/20) ASDis incidence, which was lower in the Ti groups. Guan et al. [48] and Zhao et al. [50], respectively, investigated the impact of Isobar semi-rigid systems with different ROMs on adjacent segments with distinct structures. They found that, compared to the Isobar TTL system, the Isobar EVO system demonstrates greater advantages in terms of retarding intervertebral disc degeneration in adjacent segments and grading the infiltration of paraspinal muscle in the upper adjacent segment (ASDeg). And concerning complications of Yang et al. [24] , Wen et al. [25] performed that one patient had cauda equina nerve root encroachment; however, the authors believe that this is due to the individual patient's anatomical abnormalities rather than the surgical instruments. Furthermore, Feng et al. [25] discovered that five patients had a screw breakage at the head–screw interface, which was less than 17 in the Ti group. Because each patient was pain free, no reoperation was required in the TTL group.

## Biomechanical studies

Nine articles [38, 51–58] regarding in vitro biomechanical tests were included in this review. Four articles [48, 54, 57, 58] were finite element studies (FEs), while others used cadaveric specimens. As a result of differences in model properties, test methods, and other influencing factors, the test results of these biomechanical studies cannot be measured across studies. Several biomechanical tests had questionable clinical applications. Many results across studies were also conflicting. We therefore only listed the parameters deemed most important to in vivo performance.

### *ROM *in vitro

The ROM of the lumbar spine was reported in 6 studies [38, 52–54, 57, 58]. All included articles concluded that the use of Isobar TTL could significantly decrease the ROM of the dynamic and fused level. Whether or not this was significantly different when other fusion and dynamic materials were used remains controversial. Cedric Barrey et al. [52] found that ROM following implantation of Isobar TTL ranged from 20 to 50% depending on the loading condition, and provide a greater control in 3D motion, especially with highest restoration observed in axial rotation, on six L2-S1 cadaveric spines in intact, injured (L4–L5 laminectomy), and restabilized (using Isobar TTL) states. However, S.N. Sangiorgio et al. [53] found that the Isobar reduced flexion by a mean of 56% ± 46%, and it was the only device to reduce axial rotation when compared to X-STOP and PercuDyn. Shih et al. [53] showed that the ROM at the implant level increased in the following order based on technique group: rigid, semi-rigid, dynamic intact, and disc degeneration. Liu et al. [54] found the ROM of the Isobar TTL was not significantly different from that of intact model in flexion, extension, lateral bending and rotation. Cedric Barrey et al. [38] also found that ROM decreased significantly following TTL and titanium rods compared to intact spine, with no significant difference between 2 groups, except in extension. Alexander Yu et al. [57] reported that no statistical difference in ROM between Isobar and titanium rods in any mode of loading.

### Intervertebral loading

Intradiscal pressure (IDP) or disc stress is thought to be connected with postoperative ASD. This relationship was reported in five studies [38, 51, 54, 56–58]. IDP significantly decreased in extension after TTL versus both intact and injured configurations. Cedric Barrey et al. [38] reported that IDP significantly decreased in extension after TTL versus both intact and injured configurations. Peak compressive stresses in the L3–4 were reduced by 1% to 2% (at 45° flexion), and the increased axial motion component of Isobar reduced peak disc stress by 8% to 9% [48]. The IDP at the implant level increased in the following order: rigid, semi-rigid, and dynamic instrumentation. In contrast, the IDP at adjacent levels increased in the reverse order [58]. Lu et al. [56] found that L3–L4 angular displacement of flexion, extension, left bending, right bending, left axial rotation, and right axial rotation were 33.0%, 20.2%, 23.9%, 18.6%, 28.8%, and 28.0%, respectively, lower than titanium rods. However, according to Alexander Yu et al. [57], there was a statistically significant increase in IDP in flexion extension movement with the Isobar compared to titanium rod, and no significance was detected during lateral bending, axial torsion, and axial compression. The IDP of the cranial adjacent level experienced 80% of the peak stresses, and these stresses were 47% lower for discs adjacent to TTL compared to those adjacent to titanium rods, which had a magnitude of 6.17 MPa [51]. Liu et al. [54] also found that adjacent disc stresses increased (L3–4) in TTL during flexion, extension, lateral bending and axial rotation were lower than titanium rods, respectively.

Facet contact force (FCF) was evaluated in two studies. Tang et al. [55] concluded that FCF in upright anterior flexion, posterior extension, lateral bending, and rotation was reduced to 4.01%, 0.74%, 3.78%, 3.45% and 19.7% of the vertical load (400 N), respectively, and the FCF was reduced to 24.99%, 17.23%, 17.0%, 18.40% and 35.99% of normal, respectively. Shih et al. [53] found that the Isobar afforded the decreased FCF, ranging from 15% (bending) to 41% (rotation), more than the Dynesys did. And for anterior column loading, three authors [54, 57, 58] found that a significant increase in interpedicular displacement during flexion–extension motion and anterior graft loading during axial compression was observed in Isobar TTL specimens. In light of Wolff’s Law, the authors concluded that an increase in anterior graft loading may enhance arthrodesis rate, which may result in speedier clinical recovery in patients implanted with Isobar TTL. Furthermore, two evaluated the stress distribution along screw–vertebral interfaces, the authors found that stresses on rod and near bone–screw interface are lower than that of the Dynesys and titanium rod [54, 58].

## Discussion

**Fig. 5:**
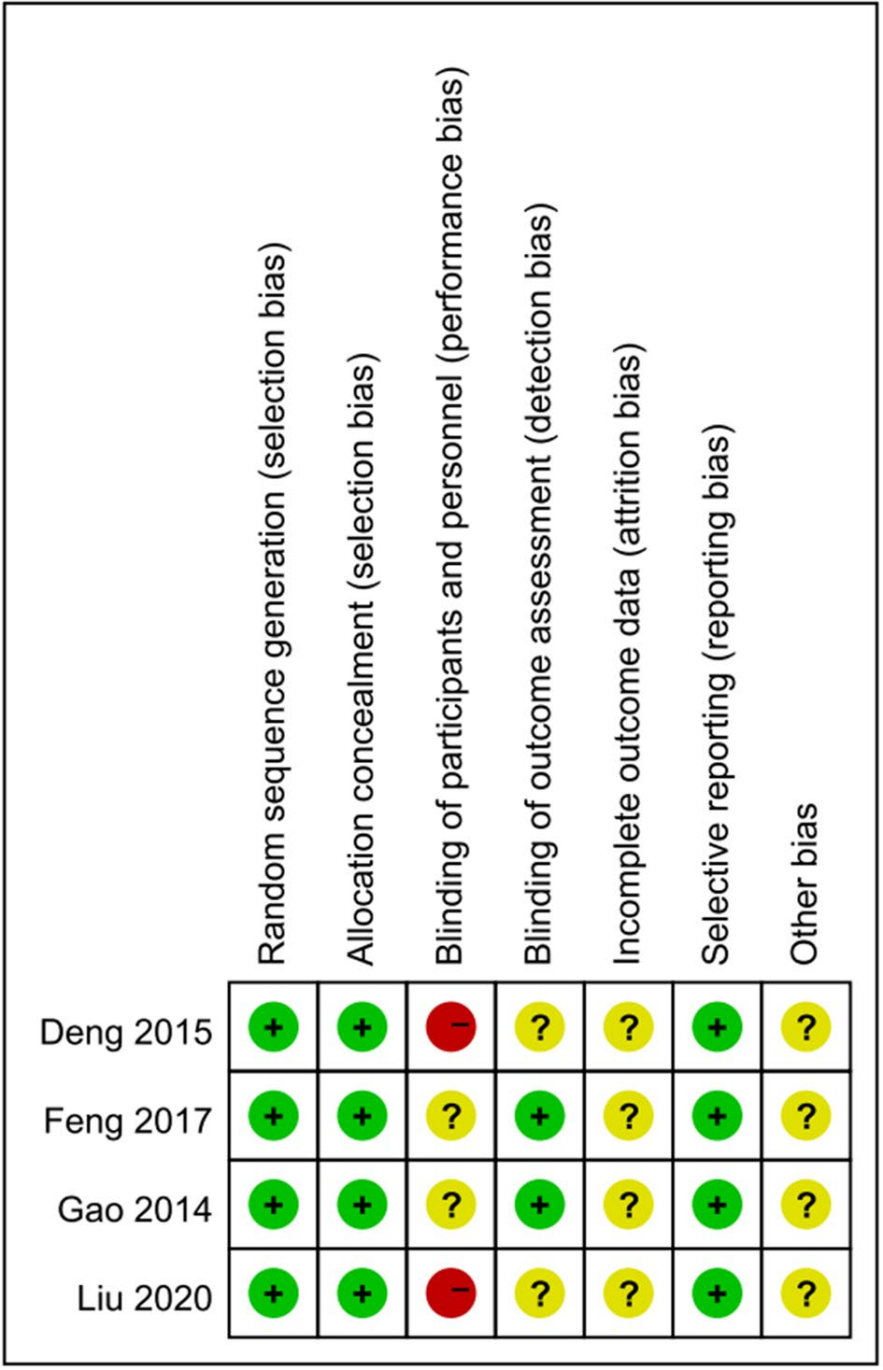
The risk of bias of including randomized controlled trials

Traditional posterior lumbar spinal fusion surgery aims to eliminate abnormal segmental movement and provide rigid stabilization for the fixed segments, resulting in a higher fusion rate and better clinical outcomes. However, some serious complications have been recorded, including ASDis, fusion failure, device failure, and persistent chronic pain [59, 60]. Fusion failure and ASDis are the most serious complications. Some authors believe that removing mechanical stresses from an interbody bone graft may result in negative bone remodeling, pseudarthrosis, and osteoporosis [61–63]. This "stress shielding" phenomena at the disc space may be caused by the excessive stiffness of conventional rigid rods. By reducing the stiffness of the instrumentation, pedicle screw-based systems (PDS) allow for load sharing between the instrumentation and the functional spine unit (FSU) at the instrumented level. As a result, to address the complications of traditional spinal fusion observed with rigid rods, dynamic instrumentation for fusion was invented in the 1990s. Using a finite element model of the spine, several authors found that posterior dynamic instrumentation, as compared to rigid instrumentation, increases the amount of load transmission between the anterior column and the interbody bone graft, preventing stress shielding (Fig. 5). This may increase osteogenesis and improve interbody fusion in accordance with Wolff’s law, which stipulates that the bone will adapt to the loads it is subjected to, i.e., the structure and shape of bone will adapt to the loading circumstances [64–66]. Overload exposes to the risk of osteonecrosis, whereas underload may result in bone graft resorption. Thus, the basic concept of dynamic fusion is fewer loads through the instruments and larger loads through the interbody bone graft while maintaining stability and load sharing (load sharing between the instrumentation and interbody bone graft, stresses reduction at bone-to-screw interface, less rigid fused segment). These advantages may result in higher fusion rates, less bone rarefaction, and fewer mechanical complications, with the ultimate objective to reduce reoperation rates. And only the semi-rigid PDS systems could logically serve for dynamic fusion since excessive flexibility provided by soft stabilization PDS devices may allow for excessive anterior loading of the interbody graft, resulting in endplate failure, subsidence, decreased fusion rates, and sagittal plane deformity (flat back) [67].

To the best of our knowledge, the Isobar semi-rigid system, which was developed from the ISOLOCK device in 1993, was one of the earliest semi-rigid rods. It has been in use for more than fifteen years in Europe, and in 1999, the FDA approved its use as a supplement to spinal fusion. G. Perrin performed the first clinical implantation of the ISOLOCK device, and in 1996 he published a study on the utility of intervertebral titanium cages with PLIF and dynamic posterior fixation in patients with lumbar degenerative disc disease and spondylolisthesis. This study reported that the fusion rate was more than 95% without any mechanical failure of the instrumentation. Unfortunately, to the best of our knowledge, there is a limited availability of prospective studies comparing rigid versus dynamic instrumentation for lumbar spine fusion. However, it is worth noting that there may be additional studies that have been published but are not widely accessible or known. Access to global information can be limited, and we acknowledge that our review may not capture all relevant research. The largest series has been reported by G. Perrin [68] (800 patients implanted with Isobar TTL), who retrospectively reported an overall fusion rate of 98% with no mechanical complications. However, because this author blended patients in his series with dynamic stabilization (non-fusion), dynamic fusion, and Hybrid surgery, the results were difficult to interpret. As a result, this study was excluded from this systematic review. Our review comprised three clinical studies, and the fusion rate ranged from 88.5% [29] to 97.9% [27, 38]. For single-segment lumbar spondylolysis with or without Meyerding grade I spondylolisthesis, two authors used posterolateral autologous isthmus grafting, and another used an anterior interbody cage for lumbar degenerative disease. The fusion rate for the posterolateral autologous isthmus grafting was 88.5% (23/26), 97.9% (48/49), and 89% (16/18) for the anterior interbody cage. However, the studies with fusion rates less than 100% were all case series with no control groups. There was no comparison of the time till unification between the groupings. Based on these facts, the data on Isobar TTL fusion rate are unclear.

### Isobar TTL could lower the probability of ASDis and ASDeg

The development of ASDis (adjacent segment disease) in patients with semi-rigid fusion technology has been under-evaluated. From the studies included in this systematic review, only one article addressed this problem. Barrey et al. [38] investigated 18 consecutives patients fused with Isobar TTL, providing a long-term (average 10.2 years) perspective on clinical outcomes. Eight patients (44.4%) presented mild radiological degenerative changes of adjacent levels lower than the literature rates that may reach 84% of radiological ASDis [69]. While the authors did not specify the standard for diagnosing ASDis, they believed that this result appeared to rely primarily on the normal aging process (mean age 56 years at surgery time), thus making the results less conclusive. It is a paradox that ASDis should be the most concerning complication of spinal fusions and also the least evaluated.

Degenerative changes in the spine, according to Kirkaldy-Willis and Farhan, can occur in three stages: (1) temporary dysfunction, (2) unstable phase, and (3) stabilization [70]. In theory, spinal fusion accelerates the transition from unstable to stabilization phase, but despite arresting instability or noxious intervertebral motions, spinal fusion does not always result in pain relief. If instability indeed was the primary cause of back pain, improvement in instrumentation techniques and fusion rates would have resulted in a proportional improvement in clinical outcomes, but clinical efficacy of fusion has plateaued over the last few decades [71]. Furthermore, fusion alters and increases load sharing mechanisms and pathways among spinal elements, as well as compensatory mobility of adjacent segments which may accelerate ASDeg (adjacent segment degeneration). Therefore, posterior dynamic stabilization devices have gained increasing popularity as an alternative surgery to fusion. However, it remains controversial whether dynamic no fusion is effective in preventing ASDeg.

In this systematic review, UCLA grading scale [72], Pfirrmann grading [73], and modified Pfirrmann grading [74] were used to assess the degeneration of adjacent segments. Six retrospective cohort study studies [23, 29, 33, 35, 36, 47] reported that the UCLA grading scale was not statistically significant between pre-op and post-op. Feng et al. [25] presented preliminary clinical outcomes of a randomized prospective control trial study including 60 patients who underwent Isobar Hybrid or titanium rod implantation for Grade I degenerative spondylolisthesis and spinal stenosis. From preoperative state to 12-month follow-up evaluation, VAS and JOA improved after surgery in both groups, but no significant difference was detected between the two groups. And UCLA of upper adjacent segment in titanium rod group was inferior to TTL group postoperatively. This result was consistent with the findings of Guan et al. [49]. A prospective randomized controlled study was conducted to compare the clinical and radiological outcomes in patients with spinal stenosis who underwent treatment with either Isobar TTL (*n* = 20) or PLIF (*n* = 21). The study did not identify any significant difference in the improvement of clinical scores between the two surgical approaches. However, based on the UCLA system, the incidence of ASDeg was observed to be 5.0% (1/20) in the Isobar TTL group and 19.0% (4/21) in the PLIF group [39]. Cao et al. [30] documented excellent patient satisfaction during the extended follow-up of 48 patients who underwent Isobar TTL with intertransverse fusion for conditions including degenerative disc disease, spinal stenosis, and instabilities. The modified Pfirrmann grading exhibited significant improvement postoperatively in both study groups. Specifically, the grade within the Isobar TTL group was notably lower than that observed in the titanium rod group. In a parallel effort, Gao et al. [45] executed a randomized controlled trial, revealing a deceleration of intervertebral disc degeneration in the Isobar TTL group at the 24-month mark post-surgery. Furthermore, 14 dynamic fixation intervertebral discs exhibited an amelioration in Pfirrmann grade. However, within the titanium rod group, 23 discs experienced a progression to a higher grade of degeneration. Huang et al. [22] introduced another distinctive assessment approach, investigating a cohort of 36 consecutive patients who had undergone single-segment non-fusion utilizing Isobar TTL. The focus of this study was the influence of lumbar nucleus pulposus volume (NPV) on the upper adjacent segmental disc, as evaluated through MRI measurements. The authors ascertained that the NPV of the upper adjacent segment demonstrated a postoperative increase, with statistical significance observed at 18, 24, 36, and 48 months. It is important to note that this study was structured as a compact case series lacking a control group, thereby rendering the findings somewhat less definitive.

Notably, when considering the broader context, studies incorporated within this systematic review indicate that Isobar TTL for non-fusion and Hybrid procedures appears to have a reduced impact on the radiological outcomes of adjacent segments compared to the conventional approach of titanium rod fusion. Furthermore, several studies within this corpus have reported superior clinical outcomes in comparison to titanium rod fusion, enhancing the appeal of Isobar TTL for surgical interventions.

### *ROM *in vivo

Mobility preservation of fixed segment and lumbar is a major concern in dynamic stabilization and no fusion surgery; however, the degree of mobility preserved varies from device to device, and the mobility of the same dynamic stabilization device in different patients varies significantly. In clinical practice, whether higher mobility of the fixed segment is better and how much mobility of the fixed segment is retained with minimal effect on the mobility of the adjacent segment are currently contentious issues. In this systematic review, the ROM in flexion/extension of fixed segment varied from 2.23° [36] to 4.04° [20, 21, 23, 26, 33–35, 37, 41, 43, 44, 46]. However, the in vitro mobility of the device is ± 2.25° which means the ROM in flexion/extension should be able to reach 4.5°, which indicates that the ROM in flexion/extension of the dynamic stabilization device in vivo is less than that in vitro. Therefore, the various data from the in vitro biomechanical study may be overall greater than that from in vivo. Moreover, based on the solid union criteria set by Suk [75], when trabecular crossing was questionable, but motion was less than 4° on flexion/extension radiographs, it was defined as solid union or probable union. As a result, Zhang et al. [21] discovered that the ROM of the dynamic fixed segment was reduced from 6.78° to 3.14° at an average of 31.9 months postoperatively and concluded that 20 cases had possible fusion and 10 cases did not, for a possible fusion rate of 66.7%. However, this subtle mobility still provides comparable or better clinical outcomes (such as VAS, ODI, and JOA scores) than conventional titanium rod fusion [24, 25, 28, 30–32, 37, 40–42, 45]. However, Deng et al. [26] discovered in an RCT that Isobar TTL provided significantly lower mobility (2.8° vs. 4.4°) and higher VAS and ODI scores at the last follow-up than Dynesys. Based on this, we hypothesize that a dynamic fixation device with greater mobility may be more beneficial for long-term clinical symptom improvement after surgery. Furthermore, the fixed segment’s mobility influenced the upper adjacent segment, and no significant change in mobility of the upper adjacent segment was found after Isobar TTL fixation in a total of seven studies [20, 33, 34, 37, 38, 40, 41], indicating that the mobility provided by Isobar TTL is effective in reducing the adjacent segment’s compensatory mobility. It is worth mentioning that the studies conducted by Guan et al. [48] and Zhao et al. [50] revealed that the Isobar EVO system with higher activity levels, in comparison to the TTL system, resulted in greater segmental mobility and reduced compensatory mobility in adjacent segments. These studies also discovered that enhancing the mobility of the fixed segment in non-fusion procedures effectively decreased degeneration in the upper adjacent segment's intervertebral disc and reduced fatty infiltration in the paraspinal muscles. As a result, this provides a conceptual pathway for subsequent research to explore whether preserving mobility could potentially delay or even prevent the degeneration of various structures in adjacent segments, such as intervertebral discs, facet joints, and vertebral body density.

By amalgamating the influences of fixed segment mobility and the conditions of the upper adjacent segments, we put forth the hypothesis that augmenting the mobility of the dynamically fixed device could potentially yield greater benefits in terms of averting degeneration and even facilitating the restoration of ASDeg.

### Pelvic parameter and complications

Sacral slope angle and lumbar lordosis correction have become important goals as these indicators have been shown to significantly improve outcomes [76]. In recent studies [24, 31, 36, 43], Isobar TTL has evolved to be a powerful approach to lumbar semi-rigid dynamic stabilization to achieve these goals. According to Huang et al. [22] and Yang et al. [24], Isobar TTL was superior to titanium rod in restoring SS angle and LL. Isobar TTL significantly improves SS angle and LL, whereas titanium rod significantly decreased sacral slope angle and lumbar lordosis. The surgical correction of the SS angle and LL holds significance in optimizing spinal alignment and balance. First, the sacral slope angle and lumbar lordosis contribute to the overall sagittal balance of the spine. Correcting these angles helps restore the natural alignment of the spine, preventing excessive forward or backward curvature. This balanced alignment is essential for maintaining proper posture and minimizing strain on spinal structures. Second, proper alignment of the SS and LL ensures optimal distribution of pressure and load across the spinal segments. This helps evenly distribute the weight-bearing forces, reducing the risk of abnormal wear and tear on intervertebral discs and facet joints. Third, the correction of these angles can lead to improved functional outcomes. It may alleviate symptoms such as back pain, improve spinal stability, and enhance the overall range of motion, allowing for better mobility and quality of life.

In addition, only two studies in our systematic review reported comparable complications with Isobar TTL and titanium rod fixation. Wen et al. [25] found that 1 patient had cauda equina nerve root encroachment in TTL Hybrid group. However, the authors did not state when the patient developed the complication, nor did they analyze the cause of the complication or explain how the patient was further managed. Another study performed by Feng et al. [28] found that the incidence of screws loosening was 2.5% (5/198) in the Isobar TTL group and 8.7% (17/196) in the rigid group. This suggests that Isobar TTL has a much lower rate of complications than titanium rod fusion and a lower chance of postoperative complications. The relatively lower occurrence of postoperative complications might be attributed to several factors. (1) Isobar non-fusion surgeries typically do not involve long-term fusion between implants and the bone, preserving more of the natural skeletal structure. This can reduce the disruption of normal bones during surgery and consequently lower the risk of postoperative complications. (2) Isobar semi-rigid system allows for some degree of skeletal movement, aiding in maintaining the physiological movement patterns. In contrast, fusion surgeries restrict certain movements, potentially leading to additional stress on surrounding structures and an increased risk of complications. (3) Isobar non-fusion surgeries typically involve smaller incisions, minimizing tissue damage and the likelihood of postoperative pain. This aids in quicker recovery and reduces the risk of complications.

### Advantages and disadvantages

**Fig. 6:**
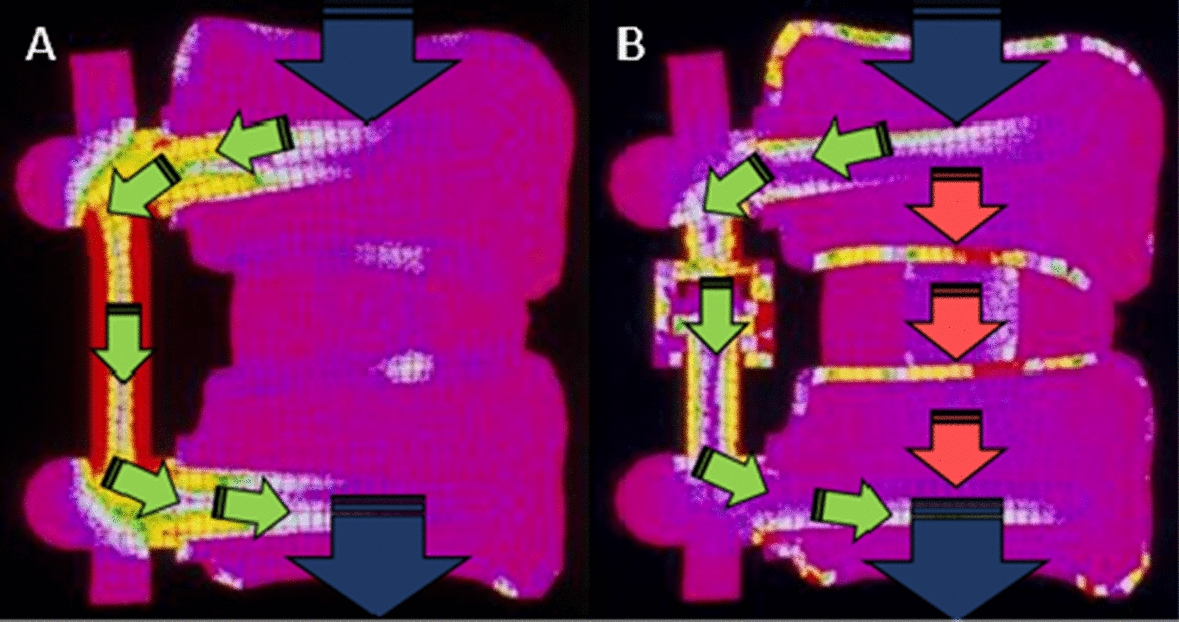
Finite element analysis illustrating load sharing phenomenon using traditional rigid system (**A**) versus Isobar semi-rigid instrumentation (**B**)

**Fig. 7:**
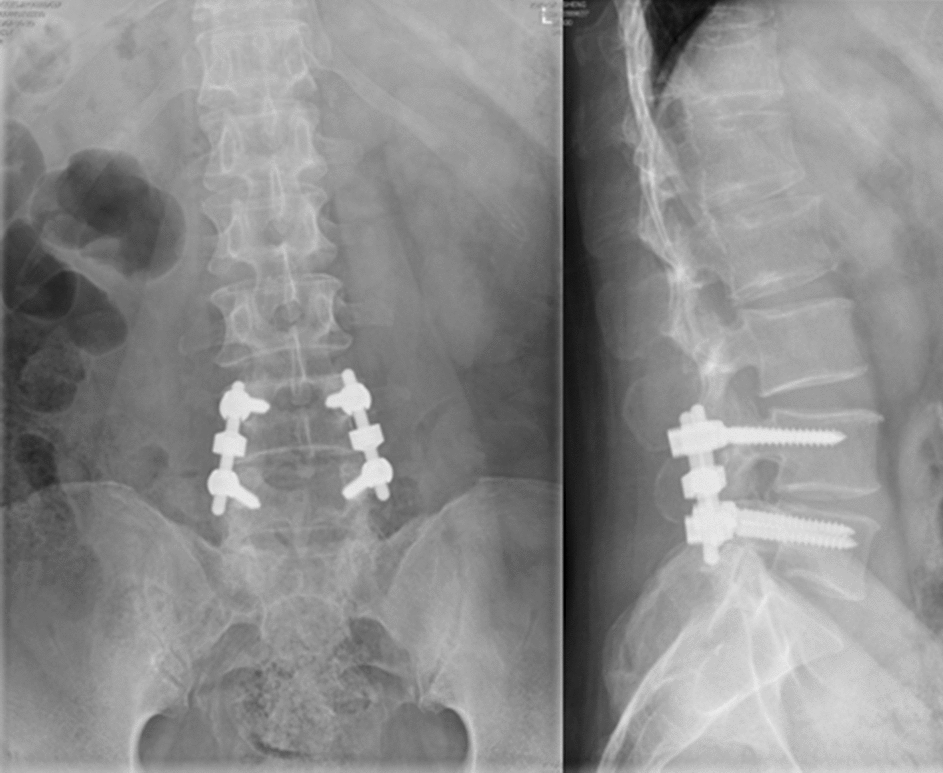
A 55-year-old male complained about his low back pain and intermittent claudication. Pre-operation X-ray indicated mild lumbar spondylolisthesis. He received decompression and semi-rigid non-fusion surgery

Theoretically, Isobar semi-rigid system has several pros and cons. In comparison to traditional titanium rods, the benefits of the Isobar semi-rigid system are as follows. (1) Isobar semi-rigid system consists of a metallic semi-rigid pedicle screw-based PDS made of titanium. It contains a damper component in its longitudinal element, a 5.5 mm titanium alloy rod. The dynamic feature of this system, represented by the damper, introduces a lowered degree of stiffness, thereby permitting controlled angular and axial micromotion. (2) It increased the amount of load transmission through the anterior column and the interbody bone graft, preventing stress shielding. This may promote osteogenesis and enhance interbody fusion in accordance with Wolff's law [61–63, 77], which states that the bone will adapt to the loads placed on it. Moreover, it concurrently diminishes stress at the rod–screw interface, thereby contributing to a decreased occurrence of hardware failure. (3) The damper provides ± 2.25° angular ROM in flexion–extension and lateral bending, no limitation in axial rotation (unconstrained). It also permits ± 0.4 mm of axial ROM. This functionality holds the potential to decrease the probability of ASDeg and ASDis. (4) Exhibiting radiolucent properties, it minimizes radiographic artifacts, thereby enabling more accurate identification of Isobar semi-rigid system (Figs. 6 and 7). (5) Regarding the surgical technique, the utilization of this implant necessitates the same procedure as fusion conducted with conventional instrumentation, involving the use of pedicle screws and rigid rods. Given the familiarity of spine surgeons with the placement of pedicle screws, there is no requirement for an additional learning curve. (6) Given the subtle differences between TTL and EVO, we believe that the Isobar system is extremely useful for investigating the effects of ROM on different structures in proximity to fixed and adjacent segments. (7) Instead of performing an intervertebral bone graft fusion, non-fusion surgery just involves decompression and fixation. As a result, the procedure takes less time and has less intraoperative blood loss.

Disadvantages include the following: (1) In the context of mobility and adjacent segments, a notable portion of studies covered in this systematic review did not differentiate and analyze data separately from the single-segment non-fusion and Hybrid surgery groups. This omission holds the potential to significantly influence the outcome of systematic review. (2) The majority of authors advocate for considering the employment of the Isobar semi-rigid system solely in the Hybrid or single-segment non-fusion approach, particularly for cases involving mild degenerative lumbar disease and spondylolisthesis of Meyerding grade I or II. This preference is mainly attributed to the absence of comprehensive data regarding the utilization of the Isobar semi-rigid system in other clinical scenarios, such as unilateral fusions or straightforward fixations accompanied by fusion. (3) An additional drawback pertains to the elevated cost associated with the implants [25]. (4) Due to the inherent limitations of non-fusion procedures, the Isobar semi-rigid fixation might not offer the comparable stability of fusion surgery for patients with severe vertebral instability. Consequently, for patients with spondylolisthesis of Meyerding grade III or higher, where stability is crucial, the Isobar system may potentially lose its clinical advantage.

It is worth mentioning that the “topping-off” technique is a concept applying dynamic or less rigid fixation such as Hybrid stabilization device (HSD) or interspinous process device (IPD) for the purpose of avoiding ASDis proximal to the fusion construct. In the absence of direct comparative studies between Isobar semi-rigid system and IPD devices, we explore the potential advantages and disadvantages of both approaches based on the available literature and clinical and biomechanical considerations. Isobar semi-rigid system, with its Hybrid stabilization approach, may offer enhanced stability compared to IPD devices, potentially reducing the risk of implant failure or migration. And the potential for Isobar semi-rigid system to achieve a more significant correction of spinal deformities or misalignments could be advantageous in specific clinical scenarios. While direct evidence is limited, there is a theoretical basis to suggest that Isobar semi-rigid system may be effective in reducing the incidence of ASDis proximal to the fusion construct, which holds clinical promise. However, Isobar semi-rigid system procedures may generally be more invasive compared to IPD device placement due to factors such as the surgical approach, tissue disruption, and potentially longer recovery times. Consideration should be given to the potential cost-related considerations associated with Isobar semi-rigid system procedures, including the cost of the Hybrid device itself and the need for specialized surgical training. Moreover, the limitation of Isobar semi-rigid system is the limited availability of long-term data on its outcomes, which can impact the ability to make definitive claims about its advantages or disadvantages.

### Surgery indication for isobar semi-rigid system

Drawing from the existing evidence within the literature, the subsequent recommendations and guidelines are put forth, taking into account specific indications. (1) The Isobar semi-rigid system could potentially be beneficial in cases where patients have herniated or significantly prolapsed discs along with spinal stenosis. (2) Spondylolisthesis involves the displacement of one vertebra over another. The Isobar semi-rigid system might be suitable for cases where this displacement is categorized as Meyerding grade I or II, providing stabilization and potentially preventing further slippage. (3) In instances where a patient requires revision surgery due to adjacent segment disease following a previous fusion surgery, the Isobar semi-rigid system might offer an alternative solution for stabilization while allowing for some degree of motion. (4) The Isobar semi-rigid system might be particularly beneficial for younger patients with degeneration of the lumbar spine. Its design may provide support while allowing for more natural movement compared to rigid fixation systems. (5) Recommended for employment in single-segment dynamic fixation, specifically for the L4/5 and L5/S1 levels. (6) When multiple spinal segments exhibit similar degrees of degeneration, a Hybrid dynamic fixation approach involving the Isobar system might be considered. This approach could provide a balance between stability and motion preservation.

It is important for medical professionals to thoroughly assess each patient's condition and medical history before making decisions about surgical interventions.

## Limitation

Following the examination of the clinical studies included in this systematic review, we observed several limitations: (1) Most authors did not report on the occurrence of ASD and the effect of adjacent segment, which is an important variable concerned with semi-rigid non-fusion. For those who did report, the follow-up period was not long enough (maximum 36 months) to make valid conclusions. (2) The sample sizes of the included studies are small, and many do not have control groups. This questions whether the study was adequately powered to fully assess all the intended outcomes. (3) Different surgery methods exist, such as pedicle screw-based anterior interbody fusions, posterolateral fusions, pedicle screw fixation without fusion, revision surgery, and Hybrid. In certain circumstances, the parameters used to evaluate the use of Isobar semi-rigid system may differ.

## Conclusion

In conclusion, Isobar semi-rigid system can be used for semi-rigid fusion, single-segment non-fusion surgery, and multilevel Hybrid for the treatment of degenerative disc disease and mild lumbar spondylolisthesis. Theoretically, Isobar semi-rigid system can improve anterior column load sharing, reducing stress shielding and appropriate fixed segmental mobility. Through its improved load sharing, decreased adjacent structure pressure, and reduced compensatory mobility of adjacent segments compared with rigid titanium rod systems, Isobar semi-rigid system reduces the symptoms of lower back pain and the incidence of ASDeg and ASDis. However, the quality of clinical studies was low, although results from mechanical studies were encouraging. More studies with better protocols, a larger sample size, and a longer follow-up time are needed.

## Methods

### Objective

The objective of this systematic review was to collect and analyze all the available information regarding the use of Isobar semi-rigid system in the semi non-fusion, Hybrid, and semi-fusion of the lumbar spine. Clinical and biomechanical data were assessed to compare the performance of Isobar system. We hoped to answer the following questions: (1) How much fixed segment ROM can Isobar semi-rigid system retain in vivo, and does ROM correlate with clinical symptoms? (2) Can Isobar semi-rigid system lower the probability of ASD? (3) What are the advantages and disadvantages of Isobar semi-rigid system? (4) what are the Indications for Isobar TTL surgery?

### Materials and methods

This systematic review is performed based on the guidance of the Preferred Reporting Items for Systematic Reviews and Meta-Analysis (PRISMA, Text 1) and Cochrane Handbook for Systematic Reviews of Interventions. No ethical approval and patient consent are required because all analyses are based on previous published studies. The review protocols were registered on PROSPERO (International Prospective Register of Systematic Reviews, ID: CRD42023457078). The specific protocol was described below.

The senior authors (Yu and Yang) preset the topic. Later, the topic was developed into detailed clinical questions described above. Discussions were held to develop the detailed eligibility criteria, search strategy, inclusion/exclusion of candidate articles, solutions when dilemma was met, etc. Webinars were held between authors from different institutions.

### Inclusion criteria

All papers that evaluated the utility and outcomes of Isobar semi-rigid system in lumbar degenerative diseases were included in this systematic review. Articles that met the following criteria were included: (1) clinical studies in which a patient cohort underwent a Isobar semi-rigid system fixation and had a specified follow-up period, (2) clinical studies evaluating clinical outcomes, such as VAS, ODI, and JOA, or radiological outcomes, and (3) biomechanical studies using cadaveric specimens or finite element models to test the strength, durability, fatigue, and other mechanical properties of Isobar semi-rigid system, range of motion analyses, disc or facet pressure analyses, changes in load sharing distribution post-fusion, and all other qualities of Isobar semi-rigid system.

### Exclusion criteria

The following types of articles were excluded: (1) articles discussing materials other than Isobar semi-rigid system, (2) articles in which the full texts were not available, (3) case reports, and (4) studies in which the indications for Isobar semi-rigid system were not specified.

### Literature search

**Fig. 8:**
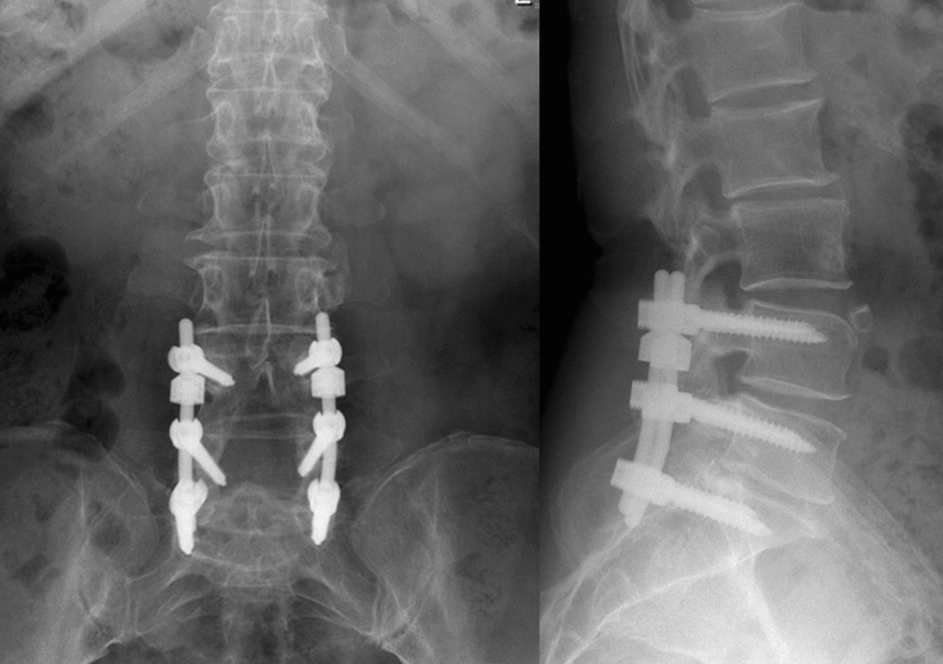
A 53-year-old female complained about his low back pain and intermittent claudication. Pre-operation X-ray indicated mild lumbar spondylolisthesis. She received decompression and Isobar TTL Hybrid surgery

A systematic computer-based retrieval was performed on the literatures published before April 1, 2023. After the eligibility criteria were established, we conducted a literature search in PubMed, Embase, Cochrane Library, China National Knowledge Infrastructure database (CNKI), Wanfang database, and China Academic Journals database (CAJ). The following search terms were used: “Isobar TTL,” “Isobar,” “dynamic stabilization,” “semi-rigid fixation,” “motion preservation,” “lumbar semi-rigid,” and “lumbar non-fusion” with the Boolean operators AND or OR. At the same time, we traced the references of the included literatures and the meta-analysis related to this research, screened, and evaluated the references to determine potential research. Further detail on the articles produced during our reference evaluation is shown in Fig. 8.

### Study selection

Two authors (Guan and Liu) were responsible for article selection and worked independently to generate their reference list. Dr. Liu is an acupuncturist, and he was invited in this study to critically and objectively select the articles that met eligibility criteria and extract the data from enrolled studies as a third-party reviewer with a less-related clinical subspecialty background. The two authors (JB.G and T.L) independently worked on reviewing the abstracts of each article, and further full-text reviews were performed if the use of Isobar TTL for lumbar degenerative diseases was discussed. If inconsistent opinions were found, an attempt at a consensus was made. If that failed, other authors were invited into the discussion until a consensus opinion evolved. Data extracted from enrolled articles are listed in Tables 3 and 4.

**Table 3:** Clinical studies using Isobar semi-rigid system

Author (year of publication)	Study design	Sample size	Surgery method	Follow-up period (months)	Major surgical indication	Parameters	Results of conclusions	Level of evidence
Qian et al. (2016) [20]	Retrospective cohort study	22	Single-level non-fusion	18 (12–27)	1.L4/5 or L5/S1 disc herniation or significant prolapse 2.Lumbar degenerative spondylolisthesis Meyerding grade I or II	VAS, JOA, ODI, ROM (FS)	VAS: 6.42 pre-op; 1.71, 1.38, and 1.37 at 1, 3, and 12 months post-op, respectively JOA: 9.54 pre-op; 21.21, 22.50, and 23.46 at 1, 3, and 12 months post-op, respectively ODI: 42.04% pre-op; 22.79, 18.63, and 15.08% at 1, 3, and 12 months post-op, respectively ROM: 3.46° at pre-op; 2.25° at last follow-up	IV
Deng et al. (2015) [21]	RCT	120	Single-level non-fusion	24	L4/5 or L5/S1 disc herniation or significant prolapse	VAS, ODI, ROM (FS)	VAS and ODI improved after surgery in both groups, but the above parameters of Isobar group higher than Dynesys group ROM: Dynesys > Isobar (4.4° vs. 2.8°)	II
Huang et al. (2016) [22]	Retrospective cohort study	36	Single-level non-fusion	48	L4/5 or L5/S1 disc herniation or significant prolapse	DH (FS), NPV of AS	The DH of FS were not significantly different between final follow-up and before surgery The NPV of AS increased post-op, showing no significant difference at 6, 12, and 18 months, but significant difference was found at 24, 36, and 48 months when compared with pre-op	IV
Tian et al. (2018) [23]	Retrospective cohort study	20	Single-level non-fusion	14.4 (12–27)	L4/5 or L5/S1 disc herniation or significant prolapse	VAS, ODI, JOA, DH (FS and AS), ROM (FS and AS), UCLA (AS)	VAS: 7.75 pre-op; 3.40, 1.60 and 0.85 at 1-, 3-, and final post-op, respectively ODI: 43.05 pre-op; 15.75, 17.19, and 12.27 at 1-, 3-, and final last follow-up JOA: 8.80 pre-op; 25.25, 27.55 and 27.65 at 1-, 3-, and last follow-up DH: FS: 13.51 mm at pre-op 12.77 mm at last follow-up AS: 15.29 mm at pre-op; 15.06 mm at last follow-up ROM: FS: 4.50° at pre-op; 2.50°at last follow-up AS: 4.80° at pre-op; 4.10° at last follow-up UCLA of AS were not significantly different pre-op and post-op	IV
Yang et al. (2012) [24]	Retrospective cohort study	98	Single-level non-fusion or Hybrid	36	1. Lumbar degenerative disease (including lumbar disc herniation, lumbar spondylotic stenosis, spondylolisthesis, and instability) 2 Spondylolisthesis (Meyerding grade I or II)	JOA, LL, SS, ASD, Complication	JOA improved in both groups, TTL group > Ti rods LL: TTL vs. Ti rods → 22.8° vs. 13.9° SS: TTL vs. Ti rods → 15.9° vs. 12.2° TTL Hybrid group: 2 ASD and 1 unfused in TTL Hybrid group Ti group: 2 screws breakage and 3 unfused and 8 ASD	III
Wen et al. (2011) [25]	Retrospective cohort study	72	Hybrid	24	1. Lumbar degenerative disease (including lumbar disc herniation, lumbar spondylotic stenosis, spondylolisthesis, and instability) 2 Lumbar degenerative spondylolisthesis Meyerding grade I or II	VAS, JOA, Complication	VAS and JOA improved after surgery in both groups, but no significant difference was detected between the 2 groups TTL Hybrid group: 1 patient had cauda equina nerve root encroachment, 1 ASD Ti group: 1 patient had screw breakage at the head–screw interface but remained pain free, 3 ASD	III
Zhang et al. (2012) [26]	Retrospective cohort study	38	Single-level non-fusion or Hybrid	27.8 (8–53)	1.Lumbar degenerative disease (including lumbar disc herniation, lumbar spondylotic stenosis, spondylolisthesis, and instability) 2.Lumbar degenerative spondylolisthesis Meyerding grade I or II	VAS, JOA, ROM (FS)	VAS: 8.20 pre-op; 1.93 final post-op JOA: 4.87 pre-op; 23.06 final post-op ROM: 3.17° at last follow-up	IV
Lu et al. (2019) [27]	Retrospective cohort study	49	Single-level fusion with isthmus bone grafting	32.12 (26–42)	Lumbar spondylolysis or with Meyerding I spondylolisthesis	VAS, ODI, Fusion rate	VAS: 6.17 pre-op; 1.76 final post-op ODI: 65.87 pre-op; 18.31 final post-op Fusion rate: 85.71% and 97.96% fusion at 6-month and 12-month post-operation	IV
Feng et al. (2017) [28]	RCT	60	Hybrid with Cage	12	1.Lumbar degenerative disease (including lumbar disc herniation, lumbar spondylotic stenosis, spondylolisthesis, and instability) 2.Lumbar degenerative Meyerding I spondylolisthesis	VAS, JOA, Complication, UCLA (AS)	VAS and JOA improved after surgery in both groups, but no significant difference was detected between the 2 groups Screws loosening: 5 (TTL 2.5%) vs. 17 (Ti rod 8.7%) UCLA of AS in Ti rod group was inferior to TTL group at 12 months postoperatively	II
Zeng et al. (2017) [29]	Retrospective cohort study	26	Single-level fusion with isthmus bone grafting	36.5 (24–60)	Lumbar spondylolysis or with Meyerding I spondylolisthesis	VAS, ODI, Fusion rate, UCLA (AS)	VAS: 5.84 pre-op; 1.46 final post-op ODI: 61.46 pre-op; 19.08 final post-op 23/26 (88.5%) fusion rate at final follow-up UCLA of AS was not significantly different between pre-op and post-op	IV
Cao et al. (2019) [30]	Retrospective cohort study	97	Hybrid with intertransverse fusion	9.2	Double-segment disc herniation combined with lumbar instability	VAS, Modified Pfirrmann grading	VAS scores improved after surgery in both groups, TTL superior to Ti rods The modified Pfirrmann grading was significantly improved postoperatively in both groups, TTL superior to Ti rods	III
Huang et al. (2012) [31]	Retrospective cohort study	74	Single-level non-fusion or Hybrid	24	1.Lumbar degenerative disease (including lumbar disc herniation, lumbar spondylotic stenosis, spondylolisthesis, and instability) 2.Lumbar degenerative spondylolisthesis Meyerding grade I or II	JOA, LA, SS ASD, Complication	JOA improved in both groups after surgery, and scores of TTL were significantly higher than that of Ti group at 6 months, 12 months, and 24 months postoperatively The LL and SS significantly different between the TTL and Ti groups, and the TTL group with a larger angle than the Ti group. (20.5° vs. 14.1°, 15.8° vs. 12.3°) TTL Hybrid group: 1ASD. Ti group: 4 ASD, 2 screws breakage and 3 segmental unfused	III
Xu et al. (2013) [32]	Retrospective cohort study	40	Single-level non-fusion or Hybrid	36 (12–46)	1.Lumbar degenerative disease (including lumbar disc herniation, lumbar spondylotic stenosis, spondylolisthesis, and instability) 2.Lumbar degenerative spondylolisthesis Meyerding grade I or II	VAS, ODI, ROM (lumbar)	VAS and ODI improved after surgery in both groups, but no significant difference was detected between the 2 groups ROM: there was not significantly difference in the TTL group between pre-op and final follow-up TTL group: 14.72° at pre-op, 13.92° at final follow-up Ti group: 13.55° at pre-op, 7.34° at final follow-up	III
Ma et al. (2019) [33]	Retrospective cohort study	40	Unilateral single-level non-fusion	53.6	L4/5 or L5/S1 disc herniation or significant prolapse	VAS, ODI, JOA, ROM, DH, UCLA, Modified Pfirrmann grading	VAS: 6.5 pre-op; 2.1 and 1.6 at 6 and 12 months post-op, respectively ODI: 71.3% pre-op; 24.8% and 20.9% at 6 and 12 months post-op, respectively JOA: 6.7 pre-op; 23.3 and 23.8 at 6 and 12 months post-op, respectively The DH was no significant difference between preoperative and postoperative (0.8 mm vs. 0.76 mm) The ROM in the AS increased significantly (2.93° vs. 5.18°), while ROM in FS decreased after surgery (2.85° vs 2.33°) The UCLA grading scale and modified Pfirrmann grading were not statistically significant between preoperative and postoperative	III
Liu et al. (2011) [34]	Retrospective cohort study	35	Single-level non-fusion or Hybrid	18 (12–36)	1.Lumbar degenerative disease (including lumbar disc herniation, lumbar spondylotic stenosis, spondylolisthesis, and instability) 2.Lumbar degenerative spondylolisthesis Meyerding grade I or II	VAS, ODI, ROM (lumbar and FS)	VAS: pre-op:6.45 last follow-up:2.12 ODI: pre-op:76.5% last follow-up:21.13% ROM (Lumbar): pre-op 14.72° vs. post-op 13.92° (*p* > 0.05) ROM (FS): pre-op 5.17° vs. post-op 4.04° (*p* > 0.05)	III
Rao et al. (2013) [35]	Retrospective cohort study	18	Single-level non-fusion or Hybrid	35 (15–52)	1.Lumbar degenerative disease (including lumbar disc herniation, lumbar spondylotic stenosis, spondylolisthesis, and instability) 2.Lumbar degenerative spondylolisthesis Meyerding grade I or II	VAS, ODI, JOA, DH (FS), ROM (FS), UCLA	VAS: pre-op:7.89 last follow-up:1.11 ODI: pre-op:24% last follow-up:6.94% JOA: pre-op:7.33 last follow-up:23.17 DH: pre-op (11.48 ± 1.70) mm vs. post-op (10.85 ± 1.32) mm (*p* > 0.05) ROM: pre-op 3.71° vs. post-op 2.72° (*p* > 0.05)	IV
Yao et al. (2017) [36]	Retrospective cohort study	20	Single-level non-fusion or Hybrid	24	1.Lumbar degenerative disease (including lumbar disc herniation, lumbar spondylotic stenosis, spondylolisthesis, and instability) 2.Lumbar degenerative spondylolisthesis Meyerding grade I or II	VAS, ODI UCLA, ROM (AS and FS), DH (AS and FS), LL, Modified Pfirrmann grading (AS and FS), Complication	VAS: pre-op:5.3 last follow-up:0.6 ODI: pre-op:54.4% last follow-up:12.9% At the latest follow-up, a significant decrease of FS: DH and ROM (from 5.26° to 2.23°) was noted; however, no significant difference in UCLA was observed compared with those before surgery AS: No significant difference was found in the DH, ROM, UCLA LL: pre-op 31.05° post-op 31.85° (*p* > 0.05) 16 patients who accepted repeat MRI at the final follow-up had no significant difference in modified Pfirrmann grading compared to that preoperatively 4/124 (3.2%) loosening screws	IV
Li et al. (2011) [37]	Retrospective cohort study	28	Single-level non-fusion	14.6 (6–24)	1.Lumbar degenerative disease (including lumbar disc herniation, lumbar spondylotic stenosis, spondylolisthesis, and instability) 2.Spondylolisthesis Meyerding grade I or II	VAS, ODI, ROM (lumbar, AS and FS)	VAS and ODI improved after surgery in both groups, but no significant difference was detected between the 2 groups In the TTL group, no significant changes of lumbar spine ROM (L2-S1) and segmental ROM (L4–5 and L3–4/L5–S1) were measured	III
Cedric Barrey et al. (2013) [38]	Retrospective cohort study	18	Single-level fusion with cage	10.2 years (7–14)	Lumbar disc herniation, lumbar spondylotic stenosis, spondylolisthesis, and instability	Fusion rate, ASD	Fusion rate: 16/18 cases (89%) 8 ASD	IV
Zhang et al. (2019) [39]	Retrospective cohort study	37	Hybrid	N/A (6–36)	1.Lumbar disc herniation, lumbar spondylotic stenosis, spondylolisthesis, and instability 2.Spondylolisthesis (Meyerding grade I or II)	VAS, ODI, DH (anterior edge and posterior edge)	VAS: pre-op:7.81 last follow-up:3.51(*p* < 0.05) ODI: pre-op:64.84% last follow-up:25.16% (*p* < 0.05) DH (anterior edge): pre-op:13.45 mm last follow-up:11.86 mm (p < 0.05) DH (posterior edge): pre-op:10.26 mm last follow-up:9.97 mm (*p* > 0.05)	IV
Liu et al. (2019) [40]	Retrospective cohort study	62	Single-level non-fusion	6	L4/5 or L5/S1 disc herniation or significant prolapse	ODI, JOA, ROM (lumbar and AS), Pfirrmann grading (AS)	ODI and JOA improved after surgery in both groups, and the ODI scores of the TTL group were significantly lower than control group and the JOA scores were significantly higher than control group The total ROM of lumbar vertebrae in the TTL group was significantly higher than that in the control group post-operation. (7.64° and 7.59° vs. 6.22° and 3.68°) And the ROM of AS in TTL group was significantly lower than that of the control group (7.30° and 7.27° vs. 7.14° and 8.26°) The Pfirrmann grading of AS in TTL group was significantly lower than that in the LD group	III
Liu et al. (2020) [41]	RCT	55	Single-level non-fusion	16.73	Singe-level disc herniation or significant prolapse	VAS, ODI, DH, ROM (FS and AS)	VAS and ODI improved after surgery in both groups, and the scores of the TTL group were significantly lower than PLED group. At final follow-up, the TTL group had significantly higher DH and ROM of FS than the PLED group (0.75 mm vs. 0.58 mm and 5.26° vs. 2.37°), and the ROM of AS was significantly lower than that of the PELD group. (7.38° vs. 9.46°)	II
Ji et al. (2020) [42]	Retrospective cohort study	41	Hybrid	22 (15–37)	Lumbar degenerative disease (including lumbar disc herniation, lumbar spondylotic stenosis, spondylolisthesis, and instability) in two consecutive segments (L3/4 and L4/5)	VAS, ODI, fusion rate, UCLA, DH(AS)	VAS and ODI improved after surgery in both groups, and difference between the two groups was not significant fusion rate: 95% (TTL) vs. 95.2% (Ti) According to the UCLA system, the incidence of ASD was 5.0% in the Isobar TTL group and 19.0% in the rigid group DH (AS): Ti group < TTL group	III
Song et al. (2021) [43]	Retrospective cohort study	15	Single-level non-fusion	45.27 ± 9.13	ASD after single-level rigid fusion	VAS, ODI, ROM (FS and AS), LL (FS and AS)	VAS: back pain: pre-op 8.07; post-op 1.07 Leg pain: pre-op 7.93; post-op 0.87 ODI: pre-op 78.65%; post-op 20.18% ROM: FS: pre-op 6.32°; post-op 3.16° (*p* < 0.05) AS: pre-op 4.84°; post-op 5.51° (*p* > 0.05) LL: FS: pre-op 27.12°; post-op 30.95° (*p* > 0.05) AS: pre-op 10.14°; post-op 11.32° (*p* > 0.05)	IV
Zhang et al. (2019) [44]	Retrospective cohort study	80	Single-level non-fusion or Hybrid	31.9 (17–45)	1.Lumbar disc herniation, lumbar spondylotic stenosis, spondylolisthesis, and instability 2.Spondylolisthesis (Meyerding grade I or II)	VAS, ODI, ROM (FS and AS)	VAS: pre-op: 9.15 last follow-up:0.24 (*p* < 0.05) ODI: pre-op:38.65% last follow-up:0.16% (*p* < 0.05) ROM: FS: pre-op 6.87°; post-op 3.14° (*p* < 0.05) AS: pre-op 4.84°; post-op 5.51° (*p* > 0.05)	IV
Gao et al. (2014) [45]	RCT	54	Hybrid	24	Lumbar degenerative spondylolisthesis Meyerding grade I or II	ODI, JOA, Pfirrmann grading (US)	ODI and JOA improved after surgery in both groups, and difference between the two groups was not significant At 24 months postoperatively, intervertebral disc degeneration in the TTL group slowed, and 14 dynamic fixation intervertebral discs showed improvement in Pfirrmann grade. However, in the Ti group, there were 23 discs which showed higher grade degeneration	II
Li et al. (2013) [46]	Retrospective cohort study	37	Hybrid	24 (12–36)	Lumbar degenerative disease (including lumbar disc herniation, lumbar spondylotic stenosis, spondylolisthesis, and instability)	ODI, VAS, ROM (FS), Pfirrmann grading (US)	VAS: back pain: pre-op 6.3; post-op 1.1 Leg pain: pre-op 7.9; post-op 1.8 ODI: pre-op 46.9%; post-op 25.4% ROM (FS): pre-op 11.2°; post-op 2.9° (*p* = 0.013)	IV
Rong et al. (2016) [47]	Retrospective cohort study	13	Single-level non-fusion	36 (24–53)	1. Spondylolysis with or without spondylolisthesis Meyerding grade I 2. Age < 25 years 3. No obvious disc degeneration at the spondylolysis level or at the adjacent level	VAS, ODI, UCLA	The mean improvement in VAS and ODI scores was 82% and 83%, respectively The AS degeneration including UCLA I grade (13 levels) and UCLA II grade (2 levels) at the final follow-up was same to pre-operation and no adjacent degenerative disease was observed	IV
Guan et al. (2022) [48]	Retrospective cohort study	80	Single-level non-fusion	52.23 ± 2.97	1.Lumbar disc herniation, lumbar spondylotic stenosis, spondylolisthesis, and instability 2.Lumbar degenerative spondylolisthesis (Meyerding I and II)	VAS, ODI, ROM (FS and AS), DH (FS and AS), UCLA (US)	No significant differences in the ODI VAS improved after surgery in both groups, the EVO group was lower compared with the TTL group ROM (FS) and DH (FS) were significantly higher in the EVO group as compared to the TTL group. (FS: 4.23° vs. 2.16°; 11.33 mm vs. 10.98) TTL group: 5 cases of UCLA grade I changes and 1 case of UCLA grade II changes occurred EVO group: 1 case of UCLA grade I change in the EVO group	III
Guan et al. (2023) [49]	Retrospective cohort study	45	Hybrid	56.09 ± 5.47	1.Mechanical low back pain, focal radiculopathy, or neurogenic claudication. 2.MRI showing nerve root compression or spinal stenosis in two segments (L3/L5 or L4/S1) with herniated or prolapsed discs (more than half of the spinal canal) 3.lumbar spondylolisthesis (Meyerding I and II)	ODI, VAS, ROM (FS and AS), DH (FS and AS), Modified Pfirrmann grading (AS and FS), LL, ASD (UCLA)	VAS improved after surgery in both groups, and difference between the two groups was not significant ODI scores, the TTL group was better than the Rigid group at 1 year after surgery and at the final follow-up ROM (FS): TTL vs. Ti rods → 3.61° vs. 1.44° ROM (US) increased in both groups, but the TTL group was lower than the Rigid group. (8.51° vs. 9.32°) The modified Pfirrmann classification of AS was significantly increased in both groups at the last follow-up LL: TTL vs. Ti rods → 38.04° vs. 37.57° The UCLA classification, the incidence of ASD was 4.2% in the TTL group and 23.8% in the Rigid group	III
Zhao et al. (2023) [50]	Retrospective cohort study	68	Single-level non-fusion	37.0 ± 19.97	1.Llumbar disc herniation, lumbar spondylotic stenosis, spondylolisthesis, and instability 2.Lumbar degenerative spondylolisthesis Meyerding grade I or II	ROM (FS and AS)	ROM: pre → post FS: TTL 9.76° → 2.60° EVO 9.90° → 5.30° PITF 9.68° → 0.77° AS: TTL 10.34° → 12.44° EVO 10.44° → 11.19° PITF 10.35° → 13.94°	III

*NPV* nucleus pulposus volume, *FS* fixation segments, *AS* adjacent segment, *LL* lumbar lordotic angle, *SS* sacral slope angle, *ASD* adjacent segment degeneration, *PLED* percutaneous endoscopic lumbar discectomy, *VAS* visual analog scale, *ODI* Oswestry disability index, JOA Japanese Orthopedic Association scoring system, *DH* disc height *UCLA* UCLA Grading Scale for Intervertebral Disc Degeneration, *ROM* range of motion, *PELD* percutaneous endoscopic lumbar discectomy, *LD* lumbar discectomy, *PITF* posterior intertransverse fusion, *N/A* not available

**Table 4:** Biomechanical studies

Author (year of publication)	Model design	Test methods	Sample size	Comparison groups	Parameters	Results of conclusions
A.E. Castellvi et al. (2005) [51]	Finite element	Finite element	N/A	Ti rods, TTL (L5–S1 was fused, and L4–5 segment was fixed with instrumentation)	Adjacent disc stresses	A 1% to 2% reduction in peak compressive stresses in the L3–4 (at 45° flexion), and the increased axial motion component of TTL reduced peak disc stress by 8% to 9%. Areas of disc tissue exposed to 80% of peak stresses of 6.17 MPa were 47% less for adjacent discs to TTL than for those adjacent to Ti rods
Cedric Barrey et al. (2010) [52]	Cadaveric lumbar spines	Displacement controlled loading	6	Intact, L4–L5 laminectomy, and L4–L5 instrumented with TTL	ROM	Flexion/extension, axial rotation and lateral bending retain 20%, 40% and 15% of intact ROM, respectively
S.N. Sangiorgio et al. (2011) [53]	Cadaveric lumbar spines	Displacement controlled loading	9	Intact, injured, X-Stop, PercuDyn	DH, ROM	With load, under maximum flexion, the Isobar increased anterior DH by 40%, compared to intact. In the neutral position with a follower load, the Isobar increased posterior DH by 40% ± 19% Comparing injured to treated specimens, the Isobar reduced flexion by a mean of 56% ± 46%. And the Isobar was the only device to reduce axial rotation
Cedric Barrey et al. (2013) [38]	Cadaveric lumbar spines	Displacement controlled loading	13	Ti rods, intact, injured (laminectomy at L4–5, L4–5 laminectomy, and partial facetectomy)	ROM, IDP	ROM decreased significantly following TTL and Ti rods compared to intact spine, with no significant difference between 2 groups, except in extension IDP significantly decreased in extension after TTL versus both intact and injured configurations
Liu et al. (2013) [54]	Finite Element	Finite Element	1	Intact, Ti rods (L4–5, L5–S1 was fused), TTL (L5–S1 was fused, and L4–5 segment was fixed with instrumentation)	ROM, stability, AS IDP, Stress distribution	The ROM of the Isobar TTL was not significantly different from that of intact model in flexion, extension, lateral bending and rotation Adjacent disc stresses increased (L3–4): TTL: flexion, extension, lateral bending, and axial rotation were 6.2%, 9.7%, 3.6%, and 3.8%, respectively. Ti rod: 8.5%, 13.5%, 4.3%, and 4.8%, respectively The stress of TTL distributed at the screw was lower than Ti rods, and the stress concentration of the fusion segment screw is more obvious
Tang et al. (2015) [55]	Cadaveric lumbar spines	Displacement controlled loading	6	Disc normal, Disc normal with Isobar TTL, Discectomy, Discectomy with TTL	FCF	FCF in upright anterior flexion, posterior extension, lateral bending, and rotation were reduced to 4.01%, 0.74%, 3.78%, 3.45%, and 19.7% of the vertical load (400 N), respectively, and the FCF was reduced to 24.99%, 17.23%, 17.0%, 18.40%, and 35.99% of normal, respectively
Lu et al. (2016) [56]	Finite Element	Finite Element	N/A	Intact, Ti rods (L4-S1)	AS angular displacement, IDP	L3–L4 angular displacement of flexion, extension, left bending, right bending, left axial rotation, and right axial rotation were 19.2%, 15.1%, 11.1%, 12.2%, 18.4%, and 22.1%, respectively, lower than Ti rods; were 11.8%, 15.7%, 6.4%, 6.5%, 11.1%, and 10.9%, respectively, higher than intact IDP:
Alexander Yu et al. (2016) [57]	Cadaveric lumbar spines	Displacement controlled loading	6	Intact, Ti rods	ROM, AL, IDP	ROM: no statistical difference in ROM between Isobar and Ti rods in any mode of loading. AL: Under axial compression, Isobar showed increased AL when compared to the Ti rods. IDP: A statistically significant increase in IDP in flexion extension movement with the Isobar compared to Ti rod, and no significance detected during lateral bending, axial torsion and axial compression
Chen et al. (2022) [58]	Finite Element	Finite Element	N/A	Dynesys, 1-level and 2-level static fixator models	ROM, IDP, FCF, stress distribution along screw–vertebral interfaces	Both the Dynesys and Isobar had better performance than the 2-level static fixator in balancing junctional problems The ROMs at L3–L4 in the Dynesys group were 15% higher in flexion, 49% higher in extension, 10% higher in lateral bending, and 8% higher in axial rotation, respectively, than those implanted with Isobar The ROMs and IDP at L2–L3 of the Isobar ranged from 2% (rotation) to 24% (extension) higher and from 2% (lateral bending) to 9% (flexion) higher, respectively, than those of the Dynesys The Isobar afforded the decreased FCF, ranging from 15% (bending) to 41% (rotation), more than the Dynesys did Stresses on rod and near bone–screw interface are lower than that of the Dynesys group

*IDP* intradiscal pressures, *ROM* range of motion, *DH* disc height, *AL* anterior column loading, *FCF* facet contact force, *AS* adjacent segment

### Quality assessment

The quality of evidence was performed on each of the selected clinical studies using rating schemes published by The Journal of Bone and Joint Surgery [18]. The quality assessment of the studies was also assessed using the Cochrane Risk-of-Bias tool for RCTs, and the Newcastle–Ottawa Scale for observational studies. There were no schemes or questionnaires available to evaluate the quality of the biomechanical studies. The rating scores were recorded on a data extraction table (Table 3). After analyzing all the included studies, recommendations were made. The quality of evidence and the strength of the recommendation scores were assessed using a modified Delphi approach by applying the Grades of Recommendation, Assessment, Development, and Evaluation (GRADE) criteria [19].

## Data Availability

The initial contributions discussed in the study are included in the article material, and further queries can be referred directly to the corresponding author.
